# Restrictive Pulmonary Disease in Diabetes Mellitus Type II Patients

**DOI:** 10.7759/cureus.23820

**Published:** 2022-04-04

**Authors:** Abdul Subhan Talpur, Kumudhavalli Kavanoor Sridhar, Khadeja Shabbir, Esinkumo E Amba-Ambaiowei, Rasha M Hasan, Zein Douedari, Nabeel Hussain, Sehrish Bader, Shahab Mirza, Farukhzad Hafizyar

**Affiliations:** 1 Medicine, Liaquat University of Medical and Health Sciences, Jamshoro, PAK; 2 Medical Microbiology, Rehoboth Diagnostic Centre, Chennai, IND; 3 Medical Microbiology, The Tamil Nadu Dr. M.G.R. Medical University, Chennai, IND; 4 Internal Medicine, Saveetha Medical College and Hospital, Chennai, IND; 5 Public Health, Southern Connecticut State University, New Haven, USA; 6 Family Medicine, College of Medicine, University of Baghdad, Baghdad, IRQ; 7 Dermatology, Aleppo University Hospital, Aleppo, SYR; 8 Basic Sciences, Saba University School of Medicine, Devens, USA; 9 Cardiovascular Medicine, Lugansk State Medical University, Luhansk, UKR; 10 Internal Medicine, Kabul University of Medical Sciences, Kabul, AFG

**Keywords:** restrictive lung disease, pulmonary function test, fvc, dmt2, diabetes

## Abstract

Background

The present study aimed to evaluate the proportion of restrictive pulmonary disease in individuals with diabetes mellitus type II patients.

Methodology

A cross-sectional study was performed at Liaquat University of Medical & Health Sciences between May 2020 and June 2021. All individuals aged between 40 and 65 years, irrespective of gender were included in the study. While those individuals with known obstructive lung diseases, blood disorders, or malignancy were excluded. Spirometry, total lung capacity (TLC), and carbon monoxide diffusing capacity (DLCO) measurements were conducted to obtain a pattern of restrictive disease in patients. Patients were divided into three main groups; i) prediabetes, ii) newly diagnosed cases of diabetes, iii) longstanding diabetes mellitus type II, and iv) control group. The parameters like the patients’ age, sex, medication, history of smoking, and cardiac diseases, among other demographics were recorded. The data collected was recorded on a predesigned proforma.

Results

The majority of the newly diagnosed cases, as well as long-standing diseases, were elderly males (p=0.014 and p<0.0001). Dyspnea was significantly correlated with longstanding diabetes mellitus type II as indicated by a higher mean score of 0.65 ± 0.10 (p=0.006). Smoking did not significantly correlate with diabetes mellitus type II. In patients with longstanding diabetes, 27 (14.4%) had a modified Medical Research Council (mMRC) score of greater than two while none of the controls had severe breathlessness. Reduced forced vital capacity (FVC) was detected in 16.0% of patients with longstanding diabetes and 12.8% in patients with newly diagnosed disease. Similar results were obtained for total lung capacity (TLC) and diffusing capacity (DLCO) (p=0.003 and p=0.02).

Conclusion

Diabetes mellitus type II is significantly associated with restrictive lung disease in patients as indicated by a high number of patients with longstanding diabetes in our study who were found to have restrictive lung disease and severe dyspnea. Screening for lung dysfunction could aid in optimum management of this debilitating disease.

## Introduction

Type II diabetes mellitus (DMT2) is a debilitating disease characterized by high blood glucose levels secondary to either insufficient insulin secretion or a combined effect of insulin resistance and its inadequate production [1]. Type II diabetes mellitus results from resistance to insulin and the inability of the body to secrete enough insulin as compensation. Type II diabetes mellitus makes up 90% of the total number of cases [2,3].

In recent years, multiple studies have been conducted that noted the worsening of pulmonary function in patients suffering from diabetes mellitus [4,5]. Factors that impact vital capacity must be evaluated as potential risk factors that might lead to the development of insulin resistance and, subsequently, diabetes mellitus [2]. According to the Fremantle Diabetes Study, of the 125 patients who visited for a follow-up for diabetes type II, 23.2% showed a forced expiratory volume in one second (FEV1) percentage-predicted value of less than 70%, while a percentage predicted value of vital capacity of less than 80%, in the absence of a diagnosed pulmonary disease [6].

Deterioration of pulmonary function in individuals with DMT2 is characterized by lower forced vital capacity (FVC), FEV1, and low peak expiratory flow (PEF) values. There was found to be a dose-response effect on pulmonary function [7]. Adult patients had a lower predicted FVC value (96 versus 103%) and FEV1 levels (92 versus 96%) as compared to patients without diabetes. Additionally, the FVC worsened more rapidly in patients with diabetes than the healthy individuals (p=0.01) [8].

Current evidence shows the effect of diabetes mellitus on pulmonary function [9]. However, no studies regarding this topic have been conducted in Pakistan, and there is insufficient data. The current study aims to assess the alterations in pulmonary function among patients of type II diabetes mellitus so that physicians can be vigilant of deterioration in respiratory function and ensure strict diabetic control. By monitoring respiratory function in diabetic patients, morbidity can be significantly reduced.

## Materials and methods

A cross-sectional study was undertaken at the Liaquat University of Medical & Health Sciences between May 2020 and June 2021. A non-randomized convenience sampling technique was employed to recruit participants. Prior to the data acquisition, ethical clearance was procured from the institutional review board (reference # LUMHS/IRB/3783).

All individuals aged between 40 and 65 years, irrespective of gender were included in the study. While those individuals with known obstructive lung diseases, blood disorders, or malignancy were excluded. Before the study was initiated, all individuals were requested to give informed written consent.

All individuals who fulfilled the eligibility criteria were screened for dyspnea via the modified Medical Research Council (mMRC) score. mMRC grades dyspnea from 0 to 4 depending on the severity of the condition. Patients were divided into three main groups according to the level of HbA1c and the duration of diabetes mellitus. Individuals having an HbA1c between 5.7% and 6.4% were labeled as “prediabetes”, those having higher than 6.5% were labeled as having diabetes - on the basis of duration of diabetes, patients were further subdivided into newly diagnosed cases (< 6 months to 2 years) and longstanding diabetes mellitus type II (> 2 years). A total of 75 healthy individuals were also enrolled to act as the “control” group. For all participants, height and weight were measured. Height was measured using a metal ruler which was fixed to the wall and the reading was recorded by a trained technician. For weight, patients were asked to remove their shoes and stand erect on a digital scale.

Spirometry (Medline 4000ml Incentive Spirometer), total lung capacity (TLC), and carbon monoxide diffusing capacity (DLCO) measurements were conducted to assess the pulmonary functions in patients. Lung function tests were performed by the same physician who remained blinded to the objectives of the study to avoid bias. TLC-P and residual volume were obtained with body plethysmography (Ganshorn PowerCube Body +, Schiller, Pakistan). Plethysmography is a device that measures changes in volume in the lungs.

A restrictive pattern was diagnosed when the following criteria were met [10]: i) FVC < lower limit normal (LLN) in adults ii) TLC-P < 80% in adults (measured by body plethysmography) iii) DLCO (<60%) iv) FEV_1_/VC (>70%)

The LLN was calculated using the online software (http://hankconsulting.com/RefCal.html).

Patient information was kept confidential, and they were explained the pros and cons of taking part in the study. The parameters like the patients’ age, sex, medication, history of smoking, and cardiac diseases, among other demographics were recorded. The data collected was recorded on a predesigned proforma. The modifying factors, including the duration of diabetes and the smoking status, were dealt with using data stratification.

Analysis of the information and statistics gathered was done using Statistical Package for the Social Sciences version 26.0 (IBM Corp., Armonk, NY). Mean and standard deviation of quantitative parameters such as age was used. Additionally, quantitative parameters such as sex and pulmonary dysfunction were expressed as frequency and percentages. Chi-square test and student t-tests were applied to ascertain the association between independent and dependent variables. Any p-value falling below 0.05 was labeled as statistically significant.

## Results

A total of 421 patients were enrolled in the study. Out of these, 112 (26.6%) were pre-diabetics, 47 (11.16%) were recently diagnosed with diabetes mellitus type II, and 187 (44.4%) had long-standing diabetes mellitus type II. Baseline characteristics of study participants are illustrated in Table 1. The majority of the recently diagnosed cases as well as longstanding cases were elderly males (p=0.014 and p<0.0001). Dyspnea was significantly correlated with longstanding diabetes mellitus type II as indicated by a higher mean score of 0.65 ± 0.10 (p=0.006). Smoking did not significantly correlate with diabetes mellitus type II. The mean weight and height of the participants were 72.7 ± 12.5 kgs and 158.8 ± 14.9 cm, respectively. The mean body mass index of the study population was 28.7 ± 3.5 kg/m^2^.

**Table 1:** Features of the study participants mMRC: modified Medical Research Council RAAS: renin-angiotensin-aldosterone system

			Diabetes Mellitus Type II		
Parameters	Control (n=75)	Prediabetic (n=112)	New diagnosis (n=47)	Longstanding (n=187)	P-value
Age, years	50.6 ± 13.4	54.2 ± 8.6	52.6 ± 14.3	65.2 ± 10.2	0.014
Gender					0.0001
Male	53 (70.7%)	68 (60.7%)	19 (40.4%)	83 (44.4%)	
Female	22 (29.3%)	44 (39.3%)	28 (59.6%)	104 (55.6%)	
mMRC score	0.12 ± 0.45	0.32 ± 0.20	0.42 ± 0.80	0.65 ± 0.10	0.006
History of smoking	8 (10.7%)	13 (11.6%)	6 (12.8%)	27 (14.4%)	0.826
History of arterial hypertension	19 (25.3%)	54 (48.2%)	13 (27.7%)	151 (80.7%)	<0.0001
History of cardiovascular disease	2 (2.7%)	23 (20.5%)	2 (4.3%)	32 (17.1%)	0.001
Medication					
Oral antidiabetics	-	-	21 (44.7%)	134 (71.7%)	<0.0001
Insulin	-	-	6 (12.8%)	58 (31%)	<0.0001
RAAS inhibitors	6 (8%)	43 (38.4%)	11 (23.4%)	116 (62%)	<0.0001
Beta-blockers	6 (8%)	25 (22.3%)	5 (10.6%)	78 (41.7%)	<0.0001
Calcium antagonists	2 (2.7%)	13 (11.6%)	2 (4.3%)	41 (21.9%)	<0.0001
Thiazide diuretics	3 (4%)	21 (18.8%)	2 (4.3%)	49 (26.2%)	<0.0001
Loop diuretics	3 (4%)	3 (2.7%)	-	19 (10.2%)	0.009
Statins	2 (2.7%)	16 (14.3%)	5 (10.64%)	68 (36.36%)	<0.0001
Acetylsalicylic acid	2 (2.7%)	18 (16.1%)	3 (6.38%)	54 (28.88%)	<0.0001

Out of 421 patients, 49 (17.1%) had restrictive lung disease as indicated by reduced TLC on body plethysmography and spirometry observations. In our study, significantly higher rates of dyspnea were observed as indicated by a higher grade of modified Medical Research Council dyspnea score (mMRC) (p<0.01). In patients with longstanding diabetes, 27 (14.4%) had an mMRC score of greater than two while none of the controls had severe breathlessness. Reduced FVC was detected in 16.0% of patients with longstanding diabetes and 12.8% in patients with newly diagnosed disease. Similar results were obtained for total lung capacity (TLC) and diffusing capacity (DLCO) (p=0.003 and p=0.02). Furthermore, in 27 (14.4%) patients with longstanding diabetes and seven patients with a recent diagnosis of DMT2, the body mass index was 30 kg/m^2^ or greater (Table 2). The mean ratio of residual volume and total lung capacity in our study was 39.6% ± 8.5.

**Table 2:** Pattern and severity of breathlessness and restrictive lung disease mMRC: modified Medical Research Council

Parameters	Control (n=75)	Prediabetic (n=112)	Recently diagnosed diabetes (n=47)	Longstanding diabetes (n=187)	P-value
mMRC score (>2)	0 (0%)	3 (2.7%)	5 (10.6%)	27 (14.4%)	<0.01
Forced vital capacity (FVC)	5 (6.7%)	8 (7.1%)	6 (12.8%)	30 (16.0%)	<0.0001
Diffusing capacity (DLCO) (<60%)	0 (0%)	5 (4.5%)	3 (6.4%)	22 (11.8%)	0.003
Total lung capacity body plethysmography (TLC-P) (<80%)	3 (4%)	10 (8.9%)	10 (21.3%)	49 (26.2%)	0.02
Body Mass Index (≥ 30 kg/m^2^)	3 (4%)	5 (4.46%)	7 (14.89%)	27 (14.4%)	0.007

## Discussion

In the present study, we found a significantly higher number of diabetic patients with restrictive patterns of pulmonary disease. Diabetes mellitus type II was found to be associated with restrictive lung disease in patients as indicated by a high frequency of individuals with longstanding diabetes.

The data observed in our study showed immense similarity with previous cross-sectional studies conducted on the same subject, which concluded that lower FVC and FEV1 values were observed in adult patients suffering from diabetes mellitus as compared to the non-diabetics, particularly in patients with a prolonged duration of diabetes mellitus requiring insulin or those who have developed complications secondary to the disease [11-13]. Results showed that in patients who did not suffer from diabetes mellitus, low FVC and FEV1 values were linked to a much higher level of fasting glucose, hyperinsulinemia, and insulin resistance.

In the present study, the body mass index of 30 kg/m^2^ or greater was associated with a restrictive lung pattern. Increased body mass index results in elevated chest wall elastic recoil, which subsequently leads to a lower end-expiratory lung volume, causing less hyperinflation. Literature reveals that obesity is significantly linked with restrictive lung disease [14].

Studies have reported data on the association between diabetes and pulmonary function. Lange et al. studied 506 adult Danish patients for more than a decade [13]. It was revealed that FVC and FEV1 were much lower than the baseline in patients with diabetes, with a difference of FVC greater than 8% among diabetics and non-diabetics. On the other hand, certain longitudinal studies found no association between diabetes and a decline in pulmonary function tests. It was found that the rate of FVC decline was 24 ml/year and 39 ml/year in diabetic women and men, respectively.

A seven-year-long study was conducted on 125 patients in Australia by Davis et al. [15]. The results showed a 68 ml/year decline in FVC and a 71 ml/year in FEV1 annually. The worsening of FVC and FEV1 was more significantly seen in patients who were reported to have a much higher baseline reading of HbA1C. However, these results were not compared with their non-diabetic counterparts. According to Litonjua et al., though patients with diabetes reported lower FEV1 and FVC levels, they only showed a 5.4 ml/year difference higher than the non-diabetic subjects [16]. As in other case-control studies, it is possible that only healthy patients with a risk of diabetes managed to complete the pulmonary function assessment. However, it has also been shown that diabetes-associated restrictive pulmonary function component is even seen in presence of associated obstructive airway diseases like asthma or chronic obstructive pulmonary disease (COPD) [17].

Even though the pathology behind the deterioration of pulmonary function in diabetic patients is unclear, it is suggested that glycosylation of the proteins in the respiratory tree and the thickening of the basal lamina could be a possible explanation [18] which may make a patient more prone to respiratory infections. It has been noted that high blood sugar levels, inflammation, and oxidative stress, which result from uncontrolled diabetes, can cause significant muscular dysfunction [19]. These adverse effects that are triggered by high levels of blood sugar are brought about due to the release of certain molecules that promote the process of inflammation [20-22].

Several studies evaluating the pulmonary function of pre-diabetic patients make such inferences more complex. Recent studies, such as the ARIC study, revealed that worsening of pulmonary function is an independent predictor of diabetes mellitus type II [22]. The present study revealed a significant cross-sectional association between diabetes mellitus and pulmonary function. The results implied that alterations in pulmonary status may occur prior to the development of diabetes and progresses during the course of the disease. It can be gathered from the findings in ARIC that mild organ failure can be linked with an alteration in gene expression, which is commonly found in conditions preceding diabetes.

The study was limited due to a population restricted to hospital patients. Additionally, a small sample size, a cross-sectional study design, and insufficient data on confounders may have reduced the precision of our results. The study could be strengthened by considering the confounders through logistic regression and a case-control study design, which would allow the comparison of diabetic and non-diabetic subjects.

## Conclusions

The study supports the idea that a deterioration in pulmonary function, especially concerning the reduction of forced vital capacity and diffusion capacity, was frequently observed in patients with a recent and longstanding diagnosis of diabetes mellitus type II. However, more research is warranted, to assess the pathology behind the association between diabetes and altered lung function. Furthermore, physicians should be vigilant about screening patients for pulmonary dysfunction especially those with long-standing type II diabetes mellitus. We recommend that diabetes mellitus type II patients should be screened for restrictive lung disease by spirometry so that early intervention could be made to reduce the morbidity.
